# Teacher Suggestion Feedback Facilitates Creativity of Students in STEAM Education

**DOI:** 10.3389/fpsyg.2021.723171

**Published:** 2021-09-01

**Authors:** Sha Shen, Saidi Wang, Yun Qi, Yanli Wang, Xiangdong Yan

**Affiliations:** ^1^College of Educational Science and Technology, Northwest Minzu University, Lanzhou, China; ^2^Gansu 24 Refractive New Media Technology Co., Ltd., Lanzhou, China; ^3^Shanghai Hui Ye (Lan Zhou) Law Office, Lanzhou, China

**Keywords:** creativity, STEAM education, teachers' formative feedback, active learning, science skills

## Abstract

This study examined the influence of the formative feedback types of teachers on creativity in Science, Technology, Engineering, Art, Mathematics (STEAM) education. Participants were 90 undergraduate students who were randomly assigned to the teacher opinions feedback group, the teacher suggestion feedback group, or the non-feedback group, and took part in three courses of STEAM education of 3D-printing technology. Before and after each course, they were asked to fill out the Eugene Creativity Test. The results showed that compare with the teacher opinions feedback group and the non-feedback group, the participants in the teacher suggestion feedback group showed a higher score on the creativity scale. This suggests that the teacher suggestion feedback can be useful for improving the creativity in STEAM education.

## Introduction

Creativity is considered to be one of the core skills of the 21st century (e.g., Gajda et al., 2017; Shin and Jang, 2017), and has been noted to be a crucial human asset necessary to deal with complex reality effectively (Corazza, 2017). Creativity is generally defined as the ability to generate new and appropriate ideas (Feist and Barron, 2003; Boden, 2004). Creativity is particularly important for college students, which is considered to be one of the necessary skills for them (e.g., Lai and Viering, 2012; Podolsky and Pogozhina, 2017; Tirri et al., 2017).

Science, Technology, Engineering, Art, Mathematics (STEAM) education, as a popular pedagogical approach to teaching, seems to have the potential to improve the creativity of students (Liliawati et al., 2018). STEAM education can be defined as “education for increasing students' interest and understanding in scientific technology and for growing STEAM literacy based on scientific technology and the ability to solve problems in the real world” (Kofac, 2017, p. 3). STEAM education combines the arts with the STEAM subjects to increase engagement, creativity, innovation, and problem-solving skills of students (e.g., Liao, 2016; National Art Education Association [NAEA], 2016), which was able to inspire learners to become more different and to be creative thinkers (Liliawati et al., 2018). Furthermore, STEAM education can make students feel at ease, can help them understand the subject and apply it to daily life (Yakman and Lee, 2012).

Some studies indicated that STEAM education enhances the creativity of students (e.g., Root-Bernstein, 2015; Liao, 2016; Oner et al., 2016; Karaca, 2017; Khamhaengpol et al., 2021). For example, Khamhaengpol et al. (2021) developed a STEAM course on nanotechnology for high school students and took 180 high school students as participants. After finishing all the courses, they measured their basic science skills, engineering design process, and creativity. The results show that the basic science skills, engineering design process, and creativity of the participants have been significantly improved.

Though researchers agree that STEAM education enhances creativity, this skill is rarely measured in studies of STEAM education (Perignat and Katz-Buonincontro, 2019). More empirical studies need to conduct for providing more evidence of the enhancing effect of STEAM education on creativity. This is an urgent question because determining whether STEAM education can enhance the creativity of students is an early and critical step for conducting STEAM education on a large scale.

One of the vital factors that appear to have the potential to influence creativity is the formative feedback of teachers (Calavia et al., 2021). “Feedback” was first applied in the fields of electronic technology and machine control, and later gradually promoted in the social sciences such as psychology and biology. In the mid-20th century, the American psychologist Skinner first proposed “procedural teaching” and pointed out that the core of procedural teaching is immediate feedback, that is after students answer questions, they should be told whether the result is correct or not in time (Rinvolucri, 1994). In the field of teaching, feedback means that in the process of teaching, teachers compare the current behavior and performance of students with the set teaching objectives and then provide students with the feedback information, so that students can improve, change, or rebuild their knowledge system according to the feedback information received (Winne and Butler, 1994). The reinforcement theory holds that reinforcement is an important reason for the change of individual behavior (Skinner, 1957; Soh, 2017). For students, the feedback received in the teaching process is the most important influencing factor in the learning process and is also the core of effective learning (Hattie and Timperley, 2007; Hattie, 2008).

The formative feedback of teachers can be divided into opinions and suggestions according to the types of feedback (Gielen et al., 2010). The opinions feedback of the teacher refers to the feedback of the teacher of the quality of the answers of students in the previous test, such as your idea is good. The suggestion feedback of the teacher refers to the further and complete suggestions of the teacher of the answers given by the students in the previous test. For example, your idea is good and innovative, and you can find relevant information online to supplement your idea. Accumulative studies have shown that formative feedback of teachers can improve the critical thinking of students (e.g., Pedrosadejesus et al., 2014; Liwen and Liu, 2018). Furthermore, there is a significant and positive relationship between critical thinking and creativity of students (e.g., Fahim and Zaker, 2014; Nosratinia and Zaker, 2014). Does this imply that the formative feedback type of teachers influences the creativity in STEAM education?

The present study used the experimental method to investigate whether the formative feedback types of teachers (teacher opinions feedback group, teacher suggestion feedback, and nonfeedback) influence the creativity of students in STEAM education. Based on previous studies, these study hypotheses that the formative feedback types of teachers influence the creativity of students in STEAM education. Specifically, the creativity of the participants in the teacher suggestion feedback group was higher than the ones in the teacher opinions feedback group and non-feedback group.

## Method

### Participants

Freshmen from a Chinese university participated in the study (*N* = 90; 48 females). They were aged 16–20 years (*M* = 17.74 SD = 0.83). According to the interviews before the formal experiment, none of the participants had participated in a similar experiment. After the experiments, all the participants received a small gift worth 50 RMB. The study protocol was approved by the local academic committee.

### Measures

#### Creativity

We used the Eugene Creativity Scale, which was compiled by Princeton Innovation Talent Research Company and has verified have good reliability and validity by domestic scholars in practice (e.g., Zhu et al., 2011; Wang, 2016). The scale includes 50 items, the first 49 items (e.g., “I do not do blind things, that is, I always have a target in mind, with the right steps to solve every problem.”) were single-choice questions of the three choices (e.g., “yes,” “no,” and “I am not sure”). The 50th is a multiple-choice question, with a total of 54 alternative words, of which only 23 words have positive weight and the rest are selected with a weight value of 0.

### Procedure

This experiment was a mixed experimental design of 3 (feedback type: teacher opinions feedback, teacher suggestion feedback, and non-feedback) ×3 (course time: the first course, the second course, and after the third course). Feedback type was the between-subjects variable, the participants were randomly assigned to the teacher opinions feedback group, the teacher suggestions feedback group, or the non-feedback group. The dependent variable was the creativity of participants which was measured by the total score on the Eugene Creativity Test.

Before the study, all the participants filled out the demographic questionnaire (gender and age) and the Eugene Creativity Test. Then, all the participants were randomly assigned to the teacher opinions feedback group, the teacher suggestions feedback group, or the non-feedback group. All the participants were given three courses of STEAM education (Table 1). In the opinions feedback situation of the teacher, the teacher only gave opinions feedback during the teaching process, such as your idea is good. In the situation of the teacher suggestion feedback, the teacher gave suggestions in the teaching process, such as your idea is good and innovative, you can find relevant materials on the Internet to supplement your idea. In the situation of the non-feedback feedback, the non-feedback is given to the students throughout the process. At the end of each course, all the participants filled out the Eugene Creativity Test.

**Table 1:** The procedure of the proposed STEAM activity.

**Week**	**Timing (minutes)**	**STEAM activity content**
1	60	Activity 1: What is 3D printing technology? In order to prepare students for this course, the teacher encourages students to think about 3D printed objects found in everyday life. After that, the teacher encouraged the students to go online and find the difference between the 3D printed objects and the traditional manufactured ones. In this activity, the students realized the important role of 3D printing technology in daily life, and knew that 3D printing is different from ordinary printing.
2	90	Activity 2: Different properties of 3D printed materialsThis activity is aimed at improving students' BBSP (Observation Skill proposed by the American Association for the Advancement of Science (AAAS, 1993).Students in each group were provided with materials commonly used in 3D printing, such as ABS plastic, PLA plastic, engineering plastic, industrial ABS material, PC material and nylon material. They observed the characteristics of different materials and discussed the uses of different materials. After that, show students how to use 3D technology and PC plastic to print a water and introduce the principle of 3D printing technology.
3	90	Activity 3: Design a 3-D item Each group of students had a period to brainstorm and share ideas among group members to design an object that could serve the public using concrete materials and 3D technology.

## Results

### Analysis of Variance for Pretests of Three Different Feedback Groups

First, ANOVA was used to calculate the differences in the creativity pretest scores of different feedback groups. The results showed that there were no significant differences in creativity between the participants in the teacher opinions feedback group (*M* = 15.47, *SD* = 5.09), the ones in the teacher suggestion feedback group (*M* = 16.83, SD = 6.66), and the ones in the nonfeedback group (*M* = 15.70, SD = 4.76) before receiving STEAM education, *F* (89) = 0.52, *p* =0.60 > 0.05.

### ANOVA of Teacher Feedback Type and Course Time on Creativity

The ANOVA results of 3 (teacher feedback type: opinions type, suggestion type, and nonfeedback) ×2 (course time: the first course, the second course, and after the third course) showed that the main effect of course was significant, *F*_(2, 174)_ = 566.85, *p* = 0 < 0.001, η*2* = 0.87. The creativity score of the participants after the third course (*M* = 52.07, SD = 0.68) was significantly higher than that after the first course (M = 23.83, SD = 0.72), *d* = 29.23, *p* = 0 < 0.001, and significantly higher than the creativity score after the second course (*M* = 35.66, SD = 0.87), *d* = 16.41, *p* = 0 < 0.001. The creativity score of the participants after the second course was significantly higher than that after the first course, *d* = 12.82, *p* = 0 < 0.001. The results showed that the creativity of participants improved significantly after receiving the STEAM course.

In addition, the main effect of teacher feedback type was significant, *F* (2, 87) = 171.95, *p* = 0 < 0.001, and η*2* = 0.80. The creativity score of the participants in the teacher suggestions feedback group (M = 51.82, SD = 0.99) was significantly higher than the ones in the teacher opinions feedback group (M = 30.47, D = 0.99), *d* = 21.36, *p* = 0 < 0.001. The creativity score of the participants in the teacher suggestions feedback group (M = 51.82, SD = 0.99) was significantly higher than the ones in the nonfeedback group (M = 28.27, SD = 0.99), *d* = 23.56, *p* = 0 < 0.001. However, there were no significant differences between the ones in the teacher opinions feedback group and the nonfeedback group, *d* = 2.20, *p* = 0.12 > 0.05. The results showed that compared with the nonfeedback and the teacher opinion feedback, the teacher suggestion feedback is more significant in promoting the creativity of students in the STEAM course.

**Figure 1:**
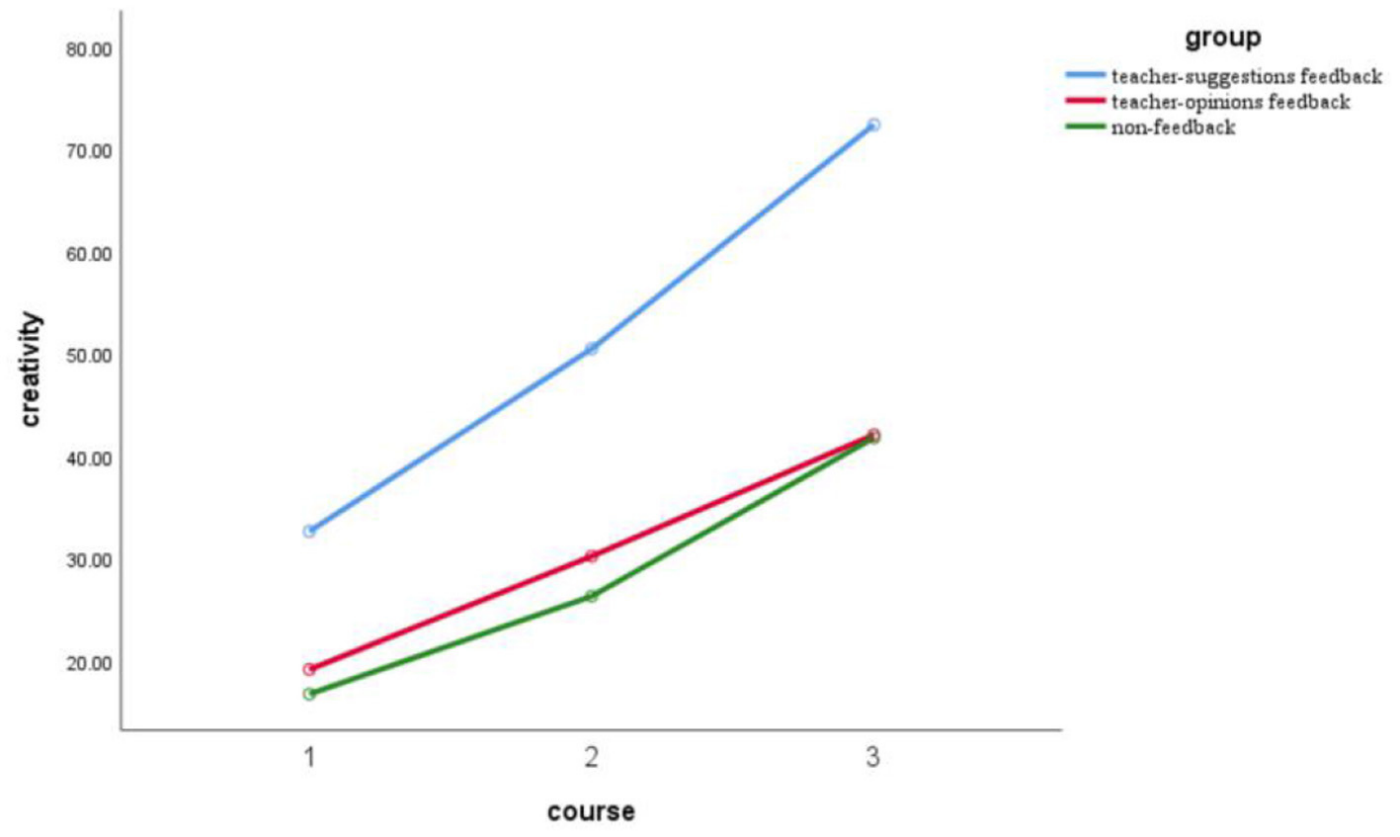
Teacher feedback type and course time on creativity.

Furthermore, the interaction between teacher feedback type and course was significant, *F* (4, 174) = 18.92, *p* = 0 < 0.05, and η*2* = 0.3 (as shown in Figure 1). After receiving the first STEAM education, the creativity score in the teacher suggestions feedback group (M = 32.63, SD = 1.79) was significantly higher than the ones in the teacher opinions feedback group (M = 19.13, SD = 0.93), *d* = 13.50, *p* = 0 < 0.001; and significantly higher than the ones in the nonfeedback group *(*M = 16.73, SD = 0.78), *d* = 15.90, *p* = 0 < 0.05. However, there were no significant differences between the ones in the teacher opinions feedback and the ones in the nonfeedback group, *d* = 2.40, *p* = 0.18 > 0.05. After receiving the second STEAM education, the creativity score in the teacher suggestions feedback group (M = 50.47, SD = 1.99) was significantly higher than the ones in the teacher opinions feedback group (M = 30.20, SD = 1.45), *d* = 26.30, *p* = 0 < 0.001; and significantly higher than the ones in the nonfeedback group (M = 26.30, SD = 0.88), *d* = 24.17, *p* = 0 < 0.05. However, there were no significant differences between the ones in the teacher opinions feedback and the ones in the nonfeedback group, *d* = 3.90, *p* = 0.07 > 0.05. After receiving the third STEAM education, the creativity score in the teacher suggestions feedback group (M = 72.37, SD = 1.31) was significantly higher than the ones in the teacher opinions feedback group *(*M = 42.07, SD=1.12), *d* = 30.30, *p* = 0 < 0.001; and significantly higher than the ones in the nonfeedback group (M = 41.77, SD = 1.10), *d* = 30.60, *p* = 0 < 0.05. However, there were no significant differences between the ones in the teacher opinions feedback and the ones in the nonfeedback group, *d* = 0.30, *p* = 0.86 > 0.05. The results showed that compared with the teacher opinions feedback and the nonfeedback, the teacher suggestion feedback can promote the creativity of the participants significantly even receiving the multiple STEAM education.

## Discussion

The present study examined whether the formative feedback types of teachers influenced the creativity of students in STEAM education. It was found that the creativity score of the participants in the teacher suggestion feedback group was significantly higher than that of the teacher opinions feedback group and the nonfeedback group. However, there were no significant differences between the ones in the teacher opinions group and the nonfeedback group. The results indicated that one of the key conditions for STEAM education to foster student creativity is to provide the students with the teacher suggestions feedback. Although researchers have admitted the potential effects of the formative feedback of teachers to creativity in STEAM education, the present study was the first attempt to examine this argument.

The present study showed that in STEAM education, the suggestion feedback of teachers promoted the creativity of students, which was consistent with the opinions of the previous studies. Studies showed that creativity is learned by the practice (Root-Bernstein, 2015), and teachers should shape the creative behavior by supporting the feedback (e.g., Cropley, 1995; Sternberg and Williams, 1996; Runco, 2014). In addition, the present study was consistent with the reinforcement theory. According to the reinforcement theory of Skinner, the behavior changes because of the reinforcement and the control of reinforcement is the control of behavior (Skinner, 1957). For learners, the feedback they receive during a course is one of the most powerful influences on their learning process (Hattie and Timperley, 2007) and central to effective learning development (Sadler, 1989). Based on a meta-analysis of more than 7,000 studies on teacher feedback in real classroom situations, Hattie and Timperley (2007) showed that the most effective feedback is to provide clues or reinforcement for the learners, associated with the correct behavior or other criteria related to the task completion. In addition, the exploratory studies of the teacher feedback in online collaborative writing tasks have shown that the learners can improve their learning if the feedback includes suggestions and questions, rather than just direct corrections.

The present study has several limitations. First, using only one creativity scale as a measure of creativity may cause the results to lack ecological validity. Future research could add other creative tasks to validate this experiment (e.g., open-ended realistic problem; Pi et al., 2019). In addition, in the process of the teaching process, the feedback of the students may also affect the results of this experiment. Future studies should examine the confounding factor to expand the understanding of the effects of teacher feedback on creativity.

This study has important theoretical significance for developing the development of an effective STEAM model. For STEAM education to develop into an effective teaching method, research is needed to understand what STEAM means in practice. Though researchers posit that STEAM education is an effective pedagogy for enhancing creativity, few empirical studies were conducted to support this notion. In addition, riches of studies indicated that arts education enhances cognitive and academic ability, whether these benefits can be transferred to STEAM education remains unclear (Perignat and Katz-Buonincontro, 2019). The present study provided evidence for the view that STEAM education is a model for enhancing creativity. In addition, this study has an important practical significance for expanding the research field of teacher feedback and creativity. Studies have examined the associations between teacher feedback and critical thinking and revealed a positive relationship between them (e.g., Pedrosadejesus et al., 2014; Liwen and Liu, 2018). However, few studies have examined the relationship between teacher feedback and creativity. To date, this is the first attempt to examine the relationships between teacher feedback and creativity under the background of STEAM education. Furthermore, this study is of great practical signicance in improving the creativity of the students. For example, in the process of STEAM teaching, we need to strengthen the suggestion feedback of teachers to promote the improvement of the creativity of students.

## Data Availability Statement

The raw data supporting the conclusions of this article will be made available by the authors, without undue reservation.

## Ethics Statement

This research was approved by the Ethics Committee of the College of Educational Science and Technology of Northwest Minzu University. All participants were volunteers who provided written informed consent, and they were told they could quit the experiment at any time if they didn't want to go on.

## Author Contributions

SS, SW, and YW proposed the original thoughts and conducted the experiments. YQ and XY collected the data and completed the article revisions. All the authors were involved in the writing of the article and approved the submitted version.

## Conflict of Interest

SW was employed by Gansu 24 refractive new media technology Co., Ltd. The remaining authors declare that the research was conducted in the absence of any commercial or financial relationships that could be construed as a potential conflict of interest.

## Publisher's Note

All claims expressed in this article are solely those of the authors and do not necessarily represent those of their affiliated organizations, or those of the publisher, the editors and the reviewers. Any product that may be evaluated in this article, or claim that may be made by its manufacturer, is not guaranteed or endorsed by the publisher.
